# Dissecting the Biology of Menstrual Cycle-Associated Breast Cancer Risk

**DOI:** 10.3389/fonc.2016.00267

**Published:** 2016-12-26

**Authors:** Vahid Atashgaran, Joseph Wrin, Simon Charles Barry, Pallave Dasari, Wendy V. Ingman

**Affiliations:** ^1^Discipline of Surgery, School of Medicine, The Queen Elizabeth Hospital, University of Adelaide, Adelaide, SA, Australia; ^2^The Robinson Research Institute, University of Adelaide, Adelaide, SA, Australia; ^3^Molecular Immunology Laboratory, Discipline of Pediatrics, University of Adelaide, North Adelaide, SA, Australia

**Keywords:** estrogen, progesterone, microenvironment, cytokine, menstrual cycle

## Abstract

Fluctuations in circulating estrogen and progesterone across the menstrual cycle lead to increased breast cancer susceptibility in women; however, the biological basis for this increased risk is not well understood. Estrogen and progesterone have important roles in normal mammary gland development, where they direct dynamic interactions among the hormonally regulated mammary epithelial, stromal, and immune cell compartments. The continuous fluctuations of estrogen and progesterone over a woman’s reproductive lifetime affect the turnover of mammary epithelium, stem cells, and the extracellular matrix, as well as regulate the phenotype and function of mammary stromal and immune cells, including macrophages and regulatory T cells. Collectively, these events may result in genome instability, increase the chance of random genetic mutations, dampen immune surveillance, and promote tolerance in the mammary gland, and thereby increase the risk of breast cancer initiation. This article reviews the current status of our understanding of the molecular and the cellular changes that occur in the mammary gland across the menstrual cycle and how continuous menstrual cycling may increase breast cancer susceptibility in women.

## Introduction

The mammary gland is an essential reproductive organ, present in females of all mammalian species, which produces milk for both nourishment and immunological protection of newborns. It is a unique organ, as the vast majority of mammary gland development occurs postnatally, during puberty, pregnancy, and the postpartum period (1). Mammary gland development is highly dependent on the actions of hormones, including estrogen and progesterone, and these endocrine factors act locally within the tissue through complex interactions with growth factors and cytokines in the mammary microenvironment (2, 3). While there has been much interest in the cellular and molecular interactions directing mammary gland development during pregnancy and postpartum involution, surprisingly little is understood of the biological mechanisms that promote development during the menstrual cycle.

A number of risk factors are associated with breast cancer, including family history of breast cancer, increasing age, and high breast density (4). There are also a number of significant risk factors associated with a woman’s reproductive history, one of which is increased number of years of menstrual cycling. A large meta-analysis demonstrated that the period of time between onset and cessation of menstrual cycling strongly correlates with increased breast cancer risk in women (5). For each year younger a girl commences menstrual cycling, there is a 5% increase in lifetime risk of breast cancer. Similarly, for each year older at the time of menopause, there is a 3.5% increased breast cancer risk (5). Studies on naturally postmenopausal women also showed increased breast cancer risk in those who had experienced greater than 490 menstrual cycles in their lifetime as compared to those with fewer or irregular cycles (6). This indicates that fluctuations in ovarian hormones associated with menstrual cycling affect breast cancer susceptibility. However, the biological basis for the link between fluctuations in ovarian hormones and increased breast cancer risk is not well understood.

The mammary gland consists of a number of different cell lineages including epithelial, hematopoietic, endothelial, and stromal (7). Epithelial cells are organized in a hierarchical manner of mammary stem cells (MaSCs), mammary progenitor cells, and committed mammary epithelial cells, including luminal and myoepithelial cells. The majority of human carcinomas originate from mammary epithelial cells (8). Immune cells, extracellular matrix (ECM), fibroblasts, and endothelial cells are all abundant in the stroma of the mammary gland (9), and their roles are mediated *via* a complex network of intracellular and extracellular signaling pathways. It is widely accepted that mutations in mammary epithelial cells are the initial drivers of tumorigenesis. However, what is increasingly appreciated are the cell-to-cell interactions between epithelia and the surrounding stroma that affect DNA mutation rate, survival of DNA-mutated cells, and development of malignancy in the breast. Fluctuations in estrogen and progesterone across the menstrual cycle affect the abundance and function of mammary epithelial cells, stromal immune cells, and the ECM, and these changes are likely to be associated with biological mechanisms that cause increased breast cancer risk associated with menstrual cycling. The focus of this review is to collate current knowledge of the molecular and cellular events that occur during hormone-mediated menstrual cycling that affect epithelial and stromal cells in the mammary gland and how these contribute to increased breast cancer risk in women.

## Biological Changes in the Breast During the Menstrual Cycle

The phases of the menstrual cycle in women are regulated by fluctuations in the pituitary gland hormones, follicle-stimulating hormone and luteinizing hormone, and the ovarian hormones, estrogen and progesterone (10). Hormonal fluctuations are cyclical in nature and occur continuously, such that each cycle merges into the next. There is also variability in cycle length (22–36 days) between different women (11). The majority of the literature on menstrual cycle-associated changes in women is consistent with mouse literature and suggests that the main proliferative phase is the mid-luteal phase of the menstrual cycle, during which time circulating progesterone and estrogen are both high and epithelial alveolar buds begin to form. The high level of mitotic activity in this phase suggests progesterone is associated with a proliferative action (12). Conversely, the late luteal phase or the menstruation phase could be considered as the regression phase of mammary gland epithelia (13). During this time, the newly formed alveolar buds undergo apoptosis and tissue remodeling occurs such that the mammary gland returns to its basic architecture ready for another menstrual cycle (1, 14).

Progesterone appears to be a key hormone in regulation of mammary gland development and regression during the cycle. A number of animal studies have demonstrated a positive correlation between the percentage of alveolar epithelial buds in the mammary gland and the concentration of serum progesterone during the ovarian cycle (15–17). Indeed, the highest percentage of alveolar epithelia were observed during the diestrus phase (mouse equivalent of luteal phase), where the concentration of serum progesterone is maximal. Although estrogen exerts proliferative effects on mammary epithelial ducts directly *via* estrogen receptors (ERs) (18), it also upregulates the expression of the progesterone receptor (PR) during the luteal phase of the cycle (19). Importantly, progesterone withdrawal is also a critical regulator of mammary gland function. Newly developed alveolar buds require continuous progesterone signaling and undergo apoptosis and tissue remodeling which returns the mammary gland to a more basic architecture when progesterone falls (17).

These cycles of hormone-driven development and regression are likely to have a significant impact on breast cancer risk, even when breast cancer arises after menopause. Nielsen et al. (20) noted that clinically occult *in situ* breast carcinomas and atypical lesions are frequent in young and middle-aged women; they may remain in the non-invasive phase for 15–20 years before they develop into invasive breast cancers. In other words, hormone-regulated cellular events that occur during premenopausal years may induce persistent changes in the developmental fate of mammary epithelial cells. Alterations in signal transduction pathways, growth factors, and cell cycle regulators (21) associated with these clinically occult cancers would increase the lifetime risk of breast cancer.

## Direct Effects of Ovarian Hormones on Tumorigenesis

The roles of estrogen and progesterone in mammary gland development have been investigated using mouse models, gene expression analysis, and normal human breast tissues. Animal studies demonstrate the critical role of estrogen in mammary tumorigenesis, as cancer initiation and development can be significantly reduced using anti-estrogenic drugs or by performing oophorectomy (22–24). This is supported in human studies wherein early ablation of the ovaries results in regression of disseminated breast cancer (25). Furthermore, use of exogenous estrogen and progesterone analogs, such as hormone replacement therapy or hormonal oral contraceptives are known to increase breast cancer risk in women (26). The primary mechanism through which exogenous and endogenous hormones are implicated in carcinogenesis is through promotion of mammary epithelial cell proliferation, which increases the chance of random genetic errors (26).

During the menstrual cycle, the highest proliferative rate of mammary epithelial cells occurs in the mid-luteal phase, as shown in premenopausal women who had highest cellular expression levels of proliferative marker Ki67 in the luteal phase versus highest expression of quiescent marker p27 in the follicular phase (27). A number of studies suggest that the mammary gland is more susceptible to carcinogenesis when there is higher proliferative activity in mammary epithelial cells (24, 28). The higher Ki67+ and the lower p27+ cell frequencies were positively associated with higher breast cancer risk among premenopausal women (27). Higher proliferative activity increases the chance of random mutations or DNA lesions (29). If the DNA-damaged cell is not repaired immediately, it will be used as a template for DNA synthesis in the next proliferative phase of the menstrual cycle, which would lead to the accumulation of faulty cells in the mammary epithelia. Polymorphisms in DNA repair genes such as BRCA2 and XRCC1, which maintain the integrity of the genome, can account for this genetic instability and the inability to repair DNA-damaged cells (30, 31). This genomic instability is favorable for premalignant cells to gain the faulty genotypes that enable tumor progression (32).

Although the role of progesterone in breast cancer etiology is controversial, it has been hypothesized that progesterone is the main driver of breast cancer risk during the menstrual cycle (33). More recently, Brisken et al. (34) proposed that repeated activation of PR signaling during the luteal phase may promote tumorigenesis in the breast. On the other hand, a recent study suggested the anti-mitogenic effects of progesterone, by inhibiting the estrogen-mediated growth of ER-positive tumors in human breast explants and cell lines (35). Overall, it seems that exposure to ovarian hormones affects cell signaling pathways and mammary progenitor cell fate. This leads to higher mitotic activity, which in turn may result in increased risk of genome instability and random genetic errors in DNA replication.

## Impact of Menstrual Cycling on MaSCs

Mammary stem cells reside within the breast tissue and support mammary gland morphogenesis during different stages of development, such as during pregnancy. These cells are capable of self-renewal divisions as well as generating various lineages of mammary epithelial cells (36, 37). With the recent advances in stem cell biology and the technical frameworks for identification of these cells, the concept that cancers originate from stem cells and that MaSCs are the targets for transformation has become a hot topic in understanding breast cancer risk.

It should be noted that only a small percentage of normal mammary epithelial cells express ER and PR, and MaSCs lack these hormone receptors. Nevertheless, they are highly responsive to and are regulated by estrogen and progesterone *via* paracrine signaling from luminal cells involving receptor activator of nuclear factor-κB ligand (RANKL), WNT, CXCL12, and amphiregulin (38–41). Investigating the phenotypes of MaSCs in the mammary gland, particularly during the menstrual cycle, can be very challenging due to their rarity and the absence of specific markers for identification of these cells (42). Although there is evidence suggesting that ovarian hormones regulate the fate of MaSCs (7), research on the link between estrogen and progesterone and these multi-potent cells during menstrual cycling is limited.

In 2009, Graham et al. (43) reported that progesterone increases proliferation of normal human mammary epithelial cells by activating DNA replication mechanisms and increasing the number of bipotent progenitor cells. However, recent studies by Lombardi et al. (44) implicate another hormone in this process. Progesterone induces normal mammary epithelial cells to secrete pituitary hormone and growth hormone, and subsequently growth hormone increases proliferation of stem and progenitor cells in the mammary gland (44). The highest levels of serum growth hormone occurs in the luteal phase, correlating with the high progesterone levels. Joshi et al. (7) observed that the abundance of MaSCs significantly increases during the diestrus phase in cycling mice as well as in ovariectomized mice treated with the combination of estradiol and progesterone.

It is speculated that the high levels of growth hormone and progesterone during the luteal phase expand the numbers and proliferation rates of undifferentiated stem cells (44). These hormones may alter the phenotypes of mammary progenitor cells and increase the likelihood of transformation of undifferentiated cells into malignancy (7). Moreover, progesterone affects both symmetric and asymmetric cell division of MaSCs by increasing the population of basal and mature mammary epithelial cells (7). An imbalance between asymmetric and symmetric stem cell divisions can occur when there is deregulation in progesterone-regulated self-renewal pathways, such as WNT and RANKL (45, 46). It may be that repetitive menstrual cycling expands the number of undifferentiated MaSCs which are more prone to oncogenic hits (39, 44), leading to an increased risk of breast cancer.

## Changes in the Immune Microenvironment During the Menstrual Cycle

Mammary gland development and function depends on dynamic interactions between hormonally responsive mammary epithelia and the immune microenvironment. Immune cells are closely associated with mammary epithelial cells (47) and contribute to a number of stages of mammary gland development. Macrophages affect development and regression of the mammary gland over the course of the cycle, and these alternating roles of macrophages may affect menstrual cycle-associated breast cancer risk, particularly during the process of mammary gland regression. Another type of immune cell that may affect cancer risk in the mammary gland during the menstrual cycle is regulatory T cells (Tregs). Although immune cells are known to have an active role in the development and function of the mammary gland, it is still not clear whether these cells affect menstrual cycle-associated breast cancer risk.

If a DNA mutation occurs, there is still a high chance that the immune system will recognize and eliminate the premalignant cell. Failure of the immune system to eliminate transformed cells throughout life can lead to cancer development. Thus, immune surveillance is a critical aspect to protect against tumorigenesis and evasion of the immune response against transformed cells is a hallmark of cancer (48). Studies on breast tumor microenvironment demonstrate that Tregs, macrophages, and other immune cells have critical roles in the immune evasion abilities of the tumor in the breast (49). The abundance and function of these cells change over the course of the menstrual cycle, potentially opening a window of breast cancer risk at specific stages of the cycle.

Fluctuations of estrogen and progesterone during the ovarian cycle influence the abundance, phenotype, and function of local macrophages in the mammary gland. Macrophages promote the proliferation of epithelial cells and formation of alveolar buds when circulating estrogen and progesterone concentrations are high and promote alveolar bud regression and tissue remodeling as circulating progesterone concentration declines (16). These processes of development and regression are associated with altered macrophage phenotype, which may affect the immune microenvironment in the mammary gland (17). The impact of hormone-regulated macrophages on breast cancer risk is not known, but may affect protection against persistence of DNA-mutated cells and tolerance to transformed cells, particularly during mammary gland regression, discussed in the next section.

Abundance of Tregs in the human blood correlates with serum concentration of estrogen; it increases during the follicular phase and decreases during the luteal phase (50). Prieto and Rosenstein (51) reported that exogenous estradiol promotes proliferation of T cell receptor-activated Tregs isolated from the blood of healthy individuals and enhances their suppressive function *in vitro*. On the other hand, progesterone regulates differentiation of naïve T cells into immune suppressive Foxp3+ Tregs in fetal cord blood and promotes immune tolerance (52). It is noteworthy that Tregs must be activated before ovarian hormones can enhance their suppressive functions (50, 51). Stimuli that activate Tregs during the menstrual cycle are not known; however, infections or altered cell signaling pathways may play a role.

Both mammary epithelial cells and immune cells secrete cytokines and chemokines, which act as intercellular mediators in the generation of immune responses. Induction of mammary epithelial differentiation is accompanied by a switch from production of Th1 cytokines (such as TNFA, IFNG, and IL12) to Th2 cytokines (such as IL13, IL10, and IL4) by mammary epithelial cells (53). Interestingly, progesterone has been shown to regulate Th1/Th2 phenotypes in the mammary gland (54) and is a potent inducer of Th2 cytokines during pregnancy (55). Th1 cytokines are more effective in producing antitumor immunity and tumor rejection, whereas Th2 cytokines are mostly produced by tumors; they induce alternatively activated macrophages and are involved in increasing humoral pro-tumorigenic responses (56, 57).

On the other hand, estradiol is shown to induce pro-inflammatory cytokine profile during the estrus phase in mice, an effect that was strongly mitigated by progesterone during other phases of the cycle (58). This inflammatory milieu may lead to tumor development and cancer progression. Thus, it seems that fluctuations of estrogen and progesterone can direct the cytokine profile of immune cells in the mammary gland. Considering the immunosuppressive roles of the immune cells in the tumor microenvironment, it is possible that these cells may weaken the mammary gland’s capability for immune detection at certain stages of the menstrual cycle and potentially affect risk of tumorigenesis in the breast.

## Increased Cancer Susceptibility During Mammary Gland Regression

The ductal and alveolar epithelial structures that form in the breast during diestrus in anticipation of pregnancy become unnecessary when the cycle progresses. These cells must be removed as the breast is remodeled during proestrus to a more basic architecture. The onset of apoptosis in mammary epithelium that occurs at the end of the luteal phase is tied to declining levels of progesterone (59). A number of animal studies have compared the sensitivity of the mammary gland to chemical carcinogens such as 9,10-dimethyl-1,2-benzanthracene (DMBA) or *N*-methyl-*N*-nitrosorea (NMU) between different phases of the ovarian cycle. Although there are some conflicting results in the literature, the majority reported that young rats exposed to chemical carcinogens at proestrus had a higher rate of mammary tumor incidence (28, 60, 61). This was accompanied by increased number of tumors, as well as shorter tumor latency than those rats injected during the metestrus or estrus phase. Proestrus is the phase in which circulating estrogen is high, and circulating progesterone is declining. This suggests that the phase of the cycle associated with mammary gland regression may be more susceptible to the initiating factors that lead to cancer than other phases of the cycle.

Macrophages are central players in the immunologically silent removal of apoptotic cells. Hodson et al. (17) reported differential percentage of murine macrophages that express cell-surface proteins NKG2D, CD204, and MHCII during different stages of the ovarian cycle, regulated by progesterone and estrogen. Macrophages present during epithelial proliferation and alveolar development during diestrus display a greater predominance of an immune surveillance phenotype, characterized by the expression of the NGK2D marker, associated with the recognition of DNA-damaged cells (17). At this stage, they are able to remove epithelial cells that have experienced replication errors. Toll-like receptors, which recognize danger-associated molecular patterns, can also participate in this activity. At proestrus, progesterone levels have declined, and this is associated with decreased NKG2D and an increase in the expression of antigen presentation receptor MHCII and scavenger receptor CD204 on macrophages, which have roles in phagocytosis and antigen presentation of dying epithelial cells (17, 62). Hence, it is suggested that fluctuations of macrophage phenotypes over the course of the ovarian cycle may regulate their capability to recognize DNA-damaged cells, phagocytose, and present antigen to generate adaptive immune responses, which may subsequently affect tumor incidence in the mammary gland (17).

Previously, it was thought that macrophages only protect the tissues from cancer, by phagocytizing the apoptotic cell debris or presenting tumor-associated antigens to T cells. However, it is now clear that these cells are also involved in breast tumorigenesis, progression, and metastasis, depending on their functional phenotype (63, 64). Macrophages are highly plastic cells that are capable of both anti-tumorigenic and pro-tumorigenic functions (56). Macrophage phenotypes and functions are heterogeneous, complex in human pathologies, and are activated by various stimuli (65). It is possible that hormonal regulations of these immune cells during menstrual cycling direct them toward a pro-tumorigenic state in which they can assist in the growth of potential tumors in the tissue.

Concomitant with the drop in progesterone is the increase in the expression and activation of TGFB1. TGFB1 has pleiotropic effects in the environment of the involuting mammary gland, first by further retarding epithelial cell proliferation and second by inducing epithelial apoptosis and causing a shift to alternative differentiation in the macrophages infiltrating the breast (62, 66). TGFB1 signaling triggers apoptosis through members of the Bcl family, leading to activation of caspase 3 with eventual nuclear condensation and DNA fragmentation (67). This process is accompanied by the release of damage-associated molecular patterns (DAMPs) or “find me” signals for immune phagocytes. Secreted DAMPs include ATP and lysophosphatidylcholine (68). In addition to DAMPs, apoptotic cells express the membrane markers phosphatidylserine (PS) and calreticulin (CRT). PS is normally found in the cell membrane on the cytoplasmic side and extracellular exposure is an early event in apoptosis. Calreticulin is found in the endoplasmic reticulum where it functions in protein folding and calcium retention in the endoplasmic reticulum (69). Apoptosis dysregulates calcium localization, leading to the release of CRT from the endoplasmic reticulum and its eventual exposure on the membrane surface (70). Membrane localization of these two molecules act as a signal of abnormal processes within the host cell and are also important in the eventual clearance of the cell by phagocytes (71, 72).

As apoptosis progresses, membrane blebbing leads to the release of exosomes. The membrane integrity deteriorates and if the cell is not cleared it will eventually become necrotic. In necrosis, the cell membrane ruptures and allows the release of pro-inflammatory cytoplasmic contents, such as Il-1 alpha and HMGB1 (73). Necrosis in breast tissue is not a desirable outcome, especially since this tissue will be exposed to hormone cycling repeatedly with attendant proliferation/apoptosis during the full extent of a woman’s reproductive life. Macrophages and other epithelial cells remove apoptotic cells from the breast before they necrose and provoke harmful inflammation. Necrosis attracts a variety of immune cells that interact to produce a vigorous response that increases the possibility of autoimmunity and carcinogenic DNA damage (74).

There is a multiplicity of mechanisms for the removal of apoptotic cells, both in terms of the target and the phagocyte. This allows for an enhanced flexibility in the host immune system and a greater likelihood an apoptotic event will be cleared and not allowed to necrose. And the key component discussed above involves PS only and does not take into account other mechanisms involving lectins, thrombospondin, or ICAM-3 (68). However, the dynamic and changing immune requirements in the breast throughout the menstrual cycle may result in mutated cells persisting from one cycle into the next, increasing the chance of tolerance of pre-cancerous cells and accumulation of further mutations that ultimately result in increased risk of cancer.

## Other Potential Cancer Pathways

The RANKL belongs to the tumor necrosis factor superfamily and acts as a paracrine modulator of progesterone action in the adult mouse mammary gland (75). RANKL also plays critical roles in progesterone-induced expansion of MaSCs and is implicated in increased breast cancer risk associated with high exposure to this hormone (76). The mRNA and protein expression of RANKL in mammary epithelium is upregulated during the luteal phase in normal breast tissues from women at standard risk of breast cancer as well as in malignant breast tissue, suggesting a role for RANKL in breast cancer initiation (40, 77). Moreover, Brisken (78) hypothesized, based on mouse model studies that repeated activation of RANKL by progesterone during the luteal phase promotes breast carcinogenesis. In short, RANKL is considered as a potential target in breast cancer treatment and prevention in premenopausal women (79).

Using next-generation whole transcriptome sequencing on 20 samples of normal human breast epithelium, Pardo et al. (40) examined the effects of hormonal fluctuations during menstrual cycle on gene expression. There were significant differences in the expression of 255 genes between the two phases of the menstrual cycle, most of which had higher expression in the luteal phase compared to the follicular phase. Genes elevated during the luteal phase include FOXM1, MYC, BCRA1, and WNT4, and are mainly involved in the cell cycle events, such as DNA replication, DNA damage response, and mitosis. Interestingly, steroid 5 alpha reductase 1 (SRD5A1) gene, which has a role in catalyzing the conversion of progesterone to 5 alpha-pregnenes mitogens *in situ* (80), was highly expressed during the luteal phase. This finding suggests that the fate of progesterone metabolism is affected during the menstrual cycle. Most of the cell cycle genes, which had higher expression in the luteal phase in this study are overexpressed in breast cancer samples (40). Hence, it is likely that the rise of progesterone during the luteal phase drives mitosis, which may increase the likelihood of genome instability and mutations in the breast and subsequently increase the risk of tumorigenesis.

In 1992, Ferguson and his colleagues noted that molecular profile of ECM in the human breast changes during the menstrual cycle *in vivo* (81). Alterations in the molecular profile of ECM would alter cell signaling and deregulate the behavior of stromal cells, which may lead to generation of a tumorigenic microenvironment (82). More recently, it was observed that gene expression of proteoglycans syndecan-1, syndecan-4, and decorin was reduced during the luteal phase in healthy breast tissues of parous women (83). Single nucleotide polymorphisms in syndeacan-1 are associated with breast cancer susceptibility (84, 85). The expression of both Syndeacan-1 and 4 is significantly correlated with human carcinoma cell proliferation (86). Reduced expression of decorin has also been observed in breast cancer tissues compared with normal tissues (87). It is suggested that lower expression of decorin weakens the ECM and is correlated with rapid progression, higher recurrence, and poor survival rate in breast cancer patients (87, 88). Proteoglycans regulate the activity of extracellular regulatory proteins in the ECM and interact with growth factors, cytokines, and chemokines (89). The direct effects of exogenous hormones on proteoglycans are not well studied. However, considering the role of proteoglycans in breast carcinomas, it is likely that hormonal regulation of these ECM components during the menstrual cycle affects cell signaling and triggers cancer pathways in the mammary gland microenvironment.

*In situ* microdialysis on normal human breast tissues revealed that the extracellular levels of vascular endothelial growth factor (VEGF), which is a potent stimulatory factor in angiogenesis, doubled during the luteal phase (90). Angiogenesis and high levels of growth factors are important factors for transformation of normal cells into malignancy (91, 92). Moreover, VEGF mRNA expression increases in breast cancer and is induced with estrogen and progestins in human breast cancer cell lines (93, 94). The higher levels of VEGF during the luteal phase suggest that there is a proangiogenic microenvironment at this time. This reflects the normal capacity of hormone-induced mammary gland to stimulate vascular growth. However, as breast cancer is an angiogenic-dependent disease (94), this proangiogenic profile might provide the essential fuel (i.e., blood supply) for the growth of potential tumor cells.

## New Directions for Breast Cancer Diagnosis and Prevention in Premenopausal Women

Understanding the biological changes that occur over the course of the menstrual cycle could lead to the development of new approaches to prevent breast cancer in premenopausal women. Studies in rodent species, discussed above, suggest that susceptibility to initiating factors that lead to cancer might be elevated during specific stages of the cycle. Alcohol consumption increases breast cancer risk, potentially through increasing circulating estrogen and enhancing estrogen responsiveness, as well as increasing production of reactive oxygen species leading to DNA damage (95, 96). Another risk factor for breast cancer is exposure to radiation. Medical imaging techniques that employ low-dose ionizing radiation, such as computed tomography, x-rays, molecular breast imaging, and mammography, can affect cancer risk (97, 98). As the susceptibility of the breast to these carcinogenic exposures might be altered by menstrual cycle stage in premenopausal women, avoidance of exposure to alcohol and ionizing radiation at specific stages of the cycle has the potential to reduce breast cancer risk.

A better understanding of how immune cell abundance and function fluctuates across the menstrual cycle may provide us with improved potential to harness the immune system to treat and prevent breast cancer. Immunotherapy and immunoprevention of cancer can involve immunization with a vaccine, passive transfer of tumor-specific antibodies, or adoptive transfer of immune cells that kill tumor cells. Changes in the types of macrophages in the breast, the cytokine microenvironment, and the phenotype of tumor-infiltrating lymphocytes across the cycle could affect the efficacy of both adaptive and humoral immune responses that recognize and eliminate tumorigenic cells (99, 100). Further studies on the effect of hormonal fluctuations on immune function could help us address such questions as how to improve immune surveillance and break the immunological tolerance induced during specific stages of the menstrual cycle.

In addition to potential for breast cancer prevention and treatment, research on the effect of menstrual cycle stage on gene expression in the breast is critical in improving the utility of PCR-based diagnostic and prognostic tests for breast cancer. The relative expression of panels of genes, employed in tests such as Oncotype DX and Prosigna, classifies tumor subtype and predicts risk of disease recurrence, in order to guide treatment decision-making (101, 102). However, such tests were developed and validated largely in postmenopausal women, and the utility of these tests in premenopausal women might be affected by fluctuating estrogen and progesterone at different stages of the menstrual cycle (103). Overall, a clearer understanding of the molecular, cellular, and immunological changes that occur in the breast during the menstrual cycle could be fundamental for improving personalized and preventive programs in breast cancer.

## Conclusion

**Figure 1:**
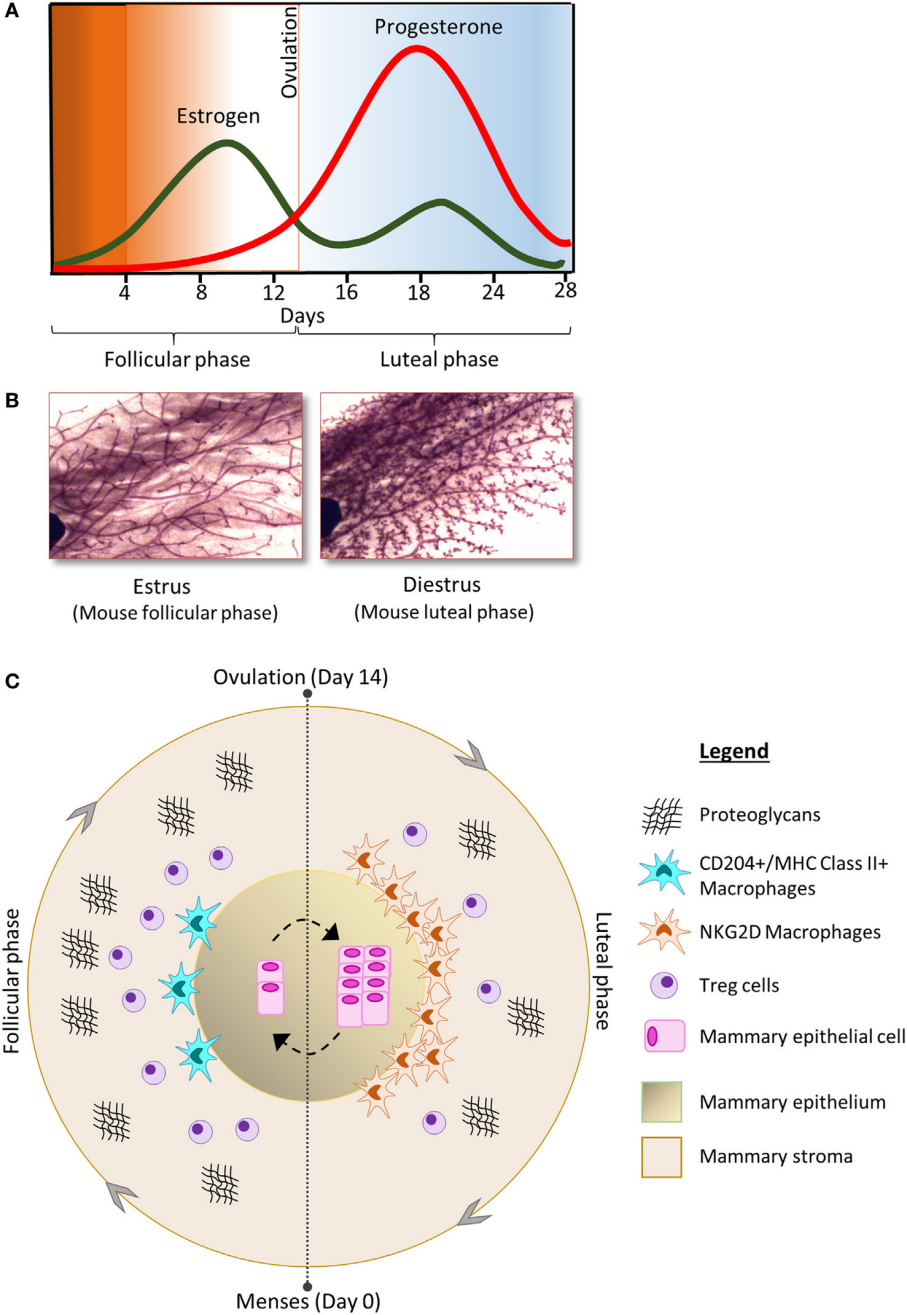
**(A)** The concentration of estrogen (green) and progesterone (red) fluctuates during the phases of the menstrual cycle and is associated with morphological changes in the cellular components of the mammary gland; shown here in carmine alum-stained mouse mammary gland whole-mount preparations **(B)** [adapted from Ref. (17) with permission]. **(C)** The follicular phase of the menstrual cycle is characterized by increase in the number of Tregs and proteoglycans compared to the luteal phase. In contrast, the luteal phase is characterized by increased numbers of mammary epithelial cells as well as increased abundance of macrophages compared to the follicular phase. The phenotype of macrophages in the mammary gland also changes throughout the cycle.

Women who undergo early menarche and/or late menopause experience higher exposure to estrogen and progesterone, and a higher number of cyclical fluctuations of these hormone during their life time. Together with the direct effects of estrogen and progesterone on cancer initiation, there are a series of coordinated events during the menstrual cycle which are regulated by these ovarian hormones which have been implicated in increased breast cancer risk. Figure 1 represents a summary of the changes in the cellular components of the mammary gland during menstrual cycling. The circle of proliferation and regression that occurs every month with each menstrual cycle affects the fate of MaSCs, which might increase the chance of random genetic errors and tumor initiation. An imbalance between mammary epithelial cell proliferation and apoptosis may provide the ideal conditions for the growth of potential tumor cells. In addition, deregulation of stromal components, such as ECM, macrophages, and Tregs, may alter the gene signaling pathways and tumor suppressor genes in the human breast, which might become persistent in some women. In addition, hormone-regulated immune cells can influence a microenvironment (e.g., by secreting cytokines and chemokines) in which immune surveillance is dampened and the breast is at increased risk of oncogenic initiation. Over time, the recurrent rise and fall in circulating estrogen and progesterone would provide the conditions for altering cell fate, increasing the risk of genome instability, and random mutations. It is a significant challenge to dissect mechanisms of menstrual cycle-associated breast cancer risk; however, the benefit will be that of a greater understanding of breast cancer susceptibility in women and the potential for discovery of new cancer biomarkers, indicators of prognosis, and therapeutic strategies to treat and prevent breast cancer.

## Author Contributions

All the authors contributed intellectually to the content and writing of the manuscript.

## Conflict of Interest Statement

The authors declare that the research was conducted in the absence of any commercial or financial relationships that could be construed as a potential conflict of interest.
